# Rutaecarpine Protects against Acetaminophen-Induced Acute Liver Injury in Mice by Activating Antioxidant Enzymes

**DOI:** 10.3390/antiox10010086

**Published:** 2021-01-10

**Authors:** Jae Ho Choi, Sun Woo Jin, Gi Ho Lee, Eun Hee Han, Yong Pil Hwang, Hye Gwang Jeong

**Affiliations:** 1 Department of Toxicology, College of Pharmacy, Chungnam National University, Daejeon 34134, Korea; chlkoala@naver.com (J.H.C.); mpassword@cnu.ac.kr (S.W.J.); ghk1900@cnu.ac.kr (G.H.L.); 2 Subtropical/Tropical Organism Gene Bank, Jeju National University, Jeju 63243, Korea; 3 Drug & Disease Target Research Team, Division of Bioconvergence Analysis, Korea Basic Science Institute (KBSI), Cheongju 28119, Korea; heh4285@kbsi.re.kr; 4 Fisheries Promotion Division, Mokpo City 58613, Korea; protoplast@hanmail.net

**Keywords:** rutaecarpine, Nrf2, antioxidant, hepatotoxicity, inflammatory cytokines

## Abstract

Rutaecarpine, an indolopyridoquinazolinone alkaloid isolated from the unripe fruit of *Evodia rutaecarpa*, is used to treat hypertension, postpartum hemorrhage, dysentery, and amenorrhea as a traditional medicine in Asia. We investigated the effect of rutaecarpine on acetaminophen-induced hepatotoxicity in mice. Rutaecarpine was administered orally daily for seven consecutive days, followed by intraperitoneal injection of acetaminophen in mice on day seven to induce hepatotoxicity. Rutaecarpine pretreatment significantly decreased acetaminophen-induced serum alanine aminotransferase (ALT)/aspartate aminotransferase (AST) activities and hepatic malondialdehyde content and prevented acetaminophen-induced hepatic glutathione depletion. Furthermore, CYP2E1 expression was decreased by rutaecarpine pretreatment in a dose-dependent manner. Rutaecarpine pretreatment inhibited acetaminophen-induced expression of inflammatory cytokines by inhibiting NF-κB activation by JNK1/2. Also, rutaecarpine pretreatment promoted Nrf2-mediated activation of the antioxidant enzymes GCLC, HO-1, and NQO1. This indicates that the protective effect of rutaecarpine during acetaminophen-induced acute liver injury is mediated by the activation of antioxidant enzymes. Therefore, rutaecarpine has a protective effect of APAP-induced liver damage.

## 1. Introduction

The liver is responsible for metabolism and detoxifying drugs. Drug-induced liver injury and liver failure lead to a high morbidity and mortality rate worldwide [1]. Most drug-induced hepatotoxicity occurs from accidental or intentional overdose of acetaminophen (APAP). APAP accounts for approximately 50% of all cases of liver injury in the United States [2]. APAP is safe at therapeutic doses and can be purchased without a prescription in combination with other drugs, but an overdose of APAP can cause serious liver damage in animals and humans [3]. APAP overdose results in approximately 80,000 emergency room visits and 30,000 hospitalizations annually in the United States [4,5].

The mechanism of APAP-induced acute liver injury involves the formation of *N*-acetyl-p-benzoquinone imine (NAPQI), a highly electrophilic metabolite, via oxidation by a cytochrome P450 enzyme (CYP2E1). NAPQI binds to cellular proteins and causes glutathione (GSH) depletion and oxidative stress, triggering signaling pathways that cause mitochondrial toxicity, thus leading to lethal hepatocyte injury. The antioxidant system is involved in maintaining the redox balance by removing reactive oxygen species (ROS) produced in the mitochondria. Antioxidant enzymes act to maintain cellular homeostasis in response to APAP-induced hepatocyte injury. As a result, the induction of antioxidant enzymes has therapeutic potential for patients with APAP-induced hepatic damage [6].

Natural substances can be effective alternative therapies for hepatic diseases [7]. In addition, there is an increasing interest in developing new and less toxic liver protectants from natural sources. Alkaloids reportedly protect against inflammation, obesity, and cancer by exerting antioxidant effects and scavenging free radicals [8]. The ability of alkaloids to provide health benefits has been evaluated extensively [9]. Rutaecarpine (Rut), an indolopyridoquinazolinone alkaloid isolated from the unripe fruit of *Evodia rutaecarpa*, is used to treat hypertension, dysentery, abdominal pain, headache, postpartum hemorrhage, and amenorrhea as a traditional medicine in Asia [10]. The pharmaceutical potential of alkaloids, in terms of inducing apoptosis of human colorectal cells and inhibiting the growth of human cancer cell lines, is established [11]. The proposed molecular mechanism is transactivation via the inhibition of the NF-κB and AP-1 signaling pathways.

We have reported that Rut protects against t-BHP-induced hepatotoxicity by upregulating antioxidant enzymes via the CaMKII-Akt and Nrf2/antioxidant responsive element (ARE) pathways [12]. However, the hepatoprotective effect of Rut has received little attention. Therefore, we evaluated the effects of Rut using an animal model of acute hepatotoxicity induced by APAP. The findings show that Rut prevented APAP-induced acute liver injury by activating antioxidant enzymes. Therefore, Rut could be useful for protecting against hepatotoxicant-induced liver injury.

## 2. Materials and Methods

### 2.1. Reagents

APAP and sodium carboxymethyl cellulose were obtained from Sigma Chemical Co. (St. Louis, MO, USA). Rut was obtained from Toronto Research Chemicals (North York, ON, Canada). DuoSet Mouse TNF-α (DY410), IL-1β (DY401), and IL-6 (DY406) enzyme-linked immunosorbent assay (ELISA) kits were obtained from R&D Systems (Minneapolis, MN, USA). Antibodies against CYP2E1, phospho-c-Jun N-terminal protein kinase (JNK) 1/2, JNK1/2, phospho-NF-κB p65, NF-κB p65, phospho-IκBα, IκBα, Nrf2, Keap1, GCLC, HO-1, NQO1, β-actin, HRP-linked anti-mouse IgG, and HRP-linked anti-rabbit IgG were purchased from Abcam, Inc. (Cambridge, MA, USA), Santa Cruz Biotechnology, Inc. (Dallas, TX, USA), and Cell Signaling Technology, Inc. (Danvers, MA, USA). PCR primers were synthesized by Bioneer Co. (Daejeon, Korea). All chemicals and reagents were of the highest commercially available grade.

### 2.2. Animals and Treatments

Specific pathogen-free 6-week-old male ICR mice were obtained from Samtako (Osan, Korea). Mice were allowed ad libitum access to Purina rodent chow (Purina, Seoul, Korea) and tap water and were maintained in a controlled environment at 22 ± 2 °C and 50 ± 5% relative humidity under a 12-h dark/light cycle, where they were acclimatized for at least 1 week before use. Sixty mice were randomly divided into the following six groups to set up a dose-response model (*n* = 10 mice/group): (1) control group, (2) 300 mg/kg APAP group, (3) 300 mg/kg APAP + 5 mg/kg Rut group, (4) 300 mg/kg APAP + 20 mg/kg Rut group, (5) 5 mg/kg Rut group, and (6) 20 mg/kg Rut group. Rut was orally administered at 5 or 20 mg/kg once daily for 7 consecutive days. Mice in the control and APAP groups were given an appropriate vehicle. After fasting for 12 h, the mice were intraperitoneally injected with APAP solution and euthanized after 8 h. Blood was collected from the vena cava, and the right liver lobe was removed, subjected to histopathological analysis, and stored at −70 °C until required for glutathione content and lipid peroxidation analyses. All experimental protocols were approved by and performed according to the rules of the Animal Ethics Committee of Chungnam National University (201903A-CNU-49).

### 2.3. Histopathological Examination

Tissues of the right lobe of the liver were sectioned, fixed in 10% neutral-buffered formalin, and subjected to hematoxylin and eosin staining (Histoire, Gyeonggi-do, Korea). Random areas of each section were viewed under a microscope at a magnification of 100×.

### 2.4. Biochemical Analysis

Hepatotoxicity was determined by measuring serum alanine aminotransferase (ALT) and aspartate aminotransferase (AST) activities, hepatic lipid peroxidation rates, and glutathione levels according to the manufacturer’s instructions. Serum ALT/AST activities were analyzed using GPT/GOT diagnostic kits (Asan Pharmaceutical Co., Seoul, Korea). Hepatic lipid peroxidation and glutathione were analyzed by measuring lipid peroxidation and using a Glutathione Colorimetric Assay kit (BioVision Inc., Milpitas, CA, USA).

### 2.5. ELISA

Serum levels of TNF-α, IL-1β, and IL-6 were measured via sandwich ELISA using R&D Systems DuoSet Mouse TNF-α (DY410), IL-1β (DY401), and IL-6 (DY406) kits according to the manufacturer’s instructions.

### 2.6. RNA Extraction and Real-Time PCR

RNA extraction, cDNA synthesis, and real-time PCR analysis were performed as described previously [13]. PCR was performed using primers for mouse TNF-α, IL-1β, IL-6, and β-actin (Table 1).

### 2.7. Western Blot

Protein extraction and western blotting were performed as described previously [13]. Protein bands were quantified using densitometry image analysis in ImageJ software (National Institutes of Health, Bethesda, MD, USA).

### 2.8. Statistical Analysis

All experiments were performed in triplicate; results are presented as the means ± SD. Statistical significance was determined by analysis of variance (ANOVA) followed by the Tukey–Kramer test, with *p* < 0.05 as the level of significance.

## 3. Results

### 3.1. Rut Pretreatment Suppressed APAP-Induced Hepatotoxicity by Attenuating CYP2E1

**Figure 1:**
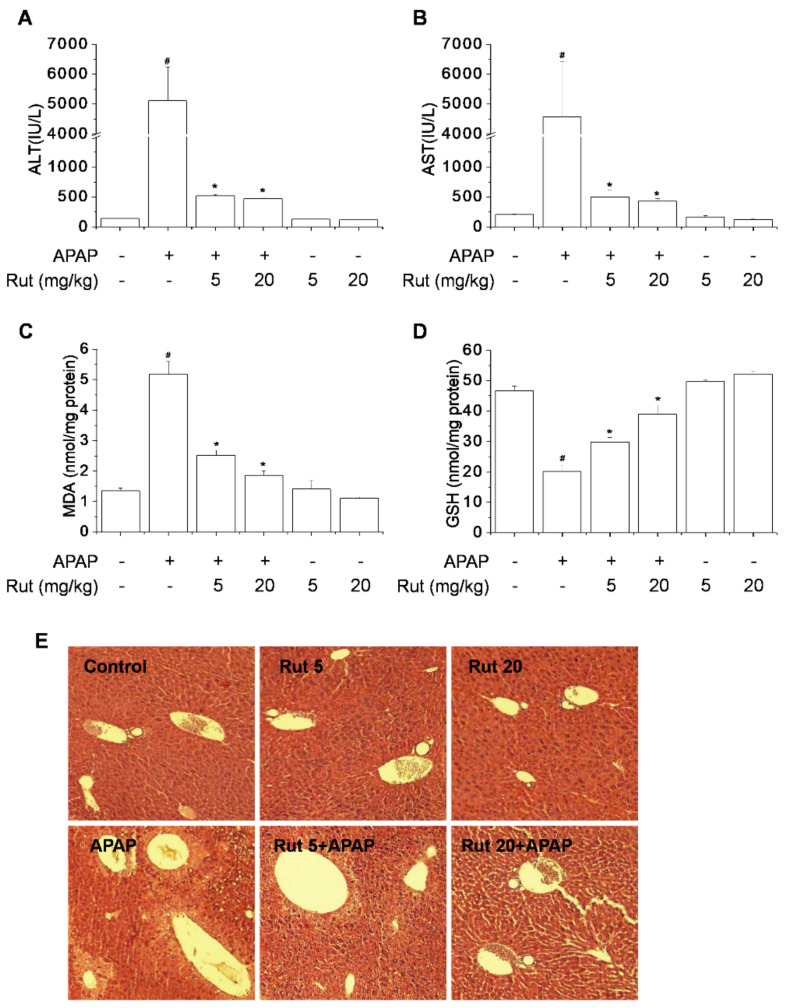
Protective effect of Rut in acetaminophen (APAP)-induced hepatotoxicity in mice. Mice were orally administered 5 or 20 mg/kg of Rut once daily for 7 consecutive days. Control and APAP-treated groups received only the appropriate vehicle orally. After fasting for 12 h, mice were intraperitoneally injected with 300 mg/kg APAP and euthanized after 8 h. Hepatotoxicity was analyzed by measuring serum alanine aminotransferase (ALT) (**A**) and aspartate aminotransferase (AST) (**B**) activities and hepatic malondialdehyde (MDA) (**C**) and glutathione (GSH) (**D**) contents. Representative hematoxylin and eosin-stained liver samples for histopathological analysis at 100× magnification (**E**). ^#^ Significantly different from the control (*p* < 0.05). * Significantly different from the APAP-treated group (*p* < 0.05).

Toxicant-induced hepatic damage is related to increased oxidative stress, which can lead to liver dysfunction. We assessed the protective effect of Rut on APAP-induced hepatotoxicity in mice using a moderate overdose of 300 mg/kg. APAP induced significant liver injury at 8 h, as indicated by the increased serum ALT and AST activities (Figure 1A,B). Also, APAP increased the hepatic malondialdehyde (MDA) content and decreased the hepatic GSH level (Figure 1C,D). Moreover, APAP caused hepatocyte necrosis in the central area of the liver (Figure 1E). These effects were dramatically reversed by Rut pretreatment in a dose-dependent manner.

**Figure 2:**
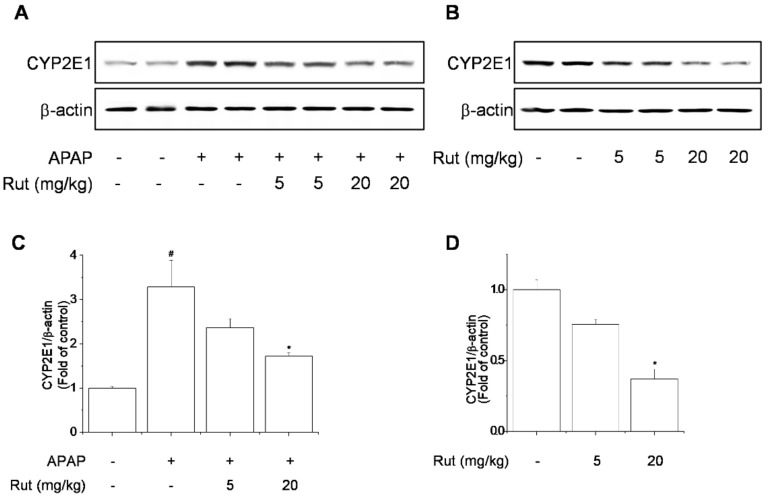
Protective effect of Rut in APAP-induced CYP2E1 expression in mice. CYP2E1 protein levels were determined using western blotting (**A**,**B**). Protein level was analyzed using ImageJ software. Relative expression of the target protein was compared using β-actin as a control (**C**,**D**). Results are indicated as means ± SD (*n* = 10). ^#^ Significantly different from the control (*p* < 0.05). * Significantly different from the APAP-treated group (*p* < 0.05).

APAP is metabolized by cytochrome P450 2E1 (CYP2E1), producing a highly reactive metabolite and causing liver damage. CYP2E1, which converts APAP to NAPQI, is responsible for APAP-mediated toxicity resulting in protein nitration and degradation [14]. Next, we evaluated the inhibitory effect of Rut on APAP-induced hepatic CYP2E1 expression. Rut pretreatment prevented APAP-induced CYP2E1 expression (Figure 2A,C). In addition, CYP2E1 expression was dose-dependently inhibited by Rut pretreatment (Figure 2B,D). These results suggest that Rut pretreatment suppressed APAP-induced hepatotoxicity by attenuating CYP2E1.

### 3.2. Rut Pretreatment Suppressed APAP-Induced Proinflammatory Cytokines by Inhibiting NF-κB Signaling

**Figure 3:**
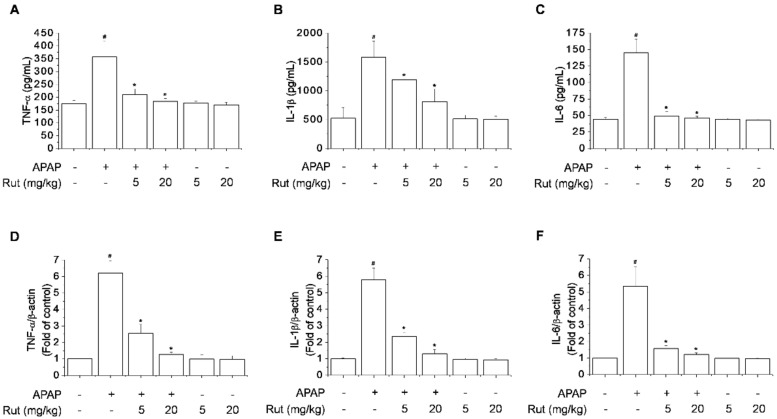
Protective effect of Rut on APAP-induced proinflammatory cytokines in mice. The mRNA expression of TNF-α (**A**), IL-1β (**B**), and IL-6 (**C**) was determined using real-time PCR and the protein levels of TNF-α (**D**), IL-1β (**E**), and IL-6 (**F**) using ELISA. Results are indicated as means ± SD (*n* = 10). ^#^ Significantly different from the control (*p* < 0.05). * Significantly different from the APAP-treated group (*p* < 0.05).

Excess proinflammatory cytokines, such as TNF-α, IL-1β, and IL-6, increase the innate immune response and cause severe liver damage following intake of toxic doses of APAP [15,16]. Furthermore, APAP-induced hepatocyte necrosis activates Kupffer cells, causing severe liver inflammation [17]. The inhibitory effect of Rut on APAP-induced hepatic mRNA expression and serum levels of proinflammatory cytokines was verified using real-time PCR and ELISA. APAP significantly increased the mRNA expression and serum levels of TNF-α, IL-1β, and IL-6 compared to the control, causing severe liver inflammation. Rut pretreatment markedly reduced these increases in a dose-dependent manner (Figure 3).

**Figure 4:**
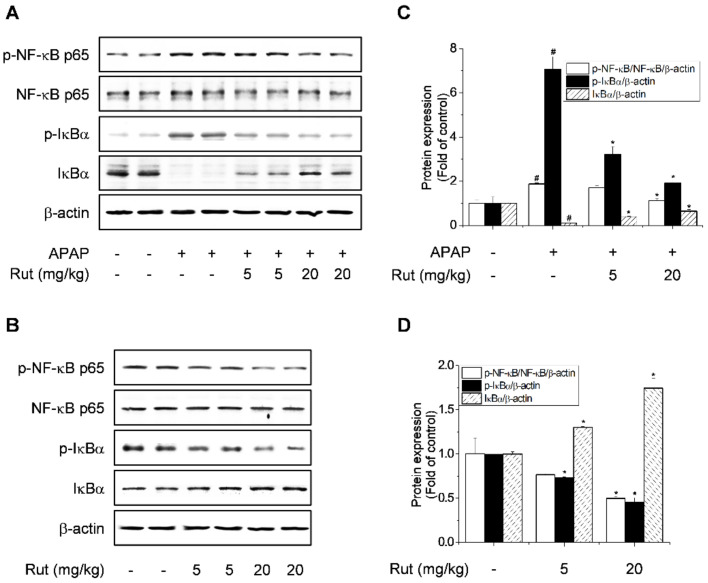
Protective effect of Rut on APAP-induced NF-κB activation in mice. Hepatic protein levels of phospho-NF-κB p65, NF-κB p65, phospho-IκBα, and IκBα were determined using western blotting (**A**,**B**). Protein level was analyzed using ImageJ software. Relative expression of the target protein was compared using β-actin as a control. (**C**,**D**) are indicated as means ± SD (*n* = 10). ^#^ Significantly different from the control (*p* < 0.05). * Significantly different from the APAP-treated group (*p* < 0.05).

Transcription factors such as NF-κB regulate the expression of genes involved in tissue damage and inflammation [18]. The inhibitory effect of Rut on APAP-induced NF-κB activation was determined by western blotting. APAP significantly induced phosphorylation of NF-κB p65 and IκBα and degradation of IκBα (Figure 4A,C); Rut pretreatment reversed these effects. In addition, Rut pretreatment inhibited the phosphorylation of NF-κB p65 and IκBα and induced the expression of IκBα in a dose-dependent manner (Figure 4B,D). Therefore, Rut pretreatment dramatically reduced the inflammatory response in APAP-treated mice, suggesting a protective effect against APAP-induced hepatic inflammation via the inhibition of NF-κB signaling pathway activation.

### 3.3. Rut Pretreatment Prevented APAP-Reduced Antioxidant Enzymes by Activating Nrf2

**Figure 5:**
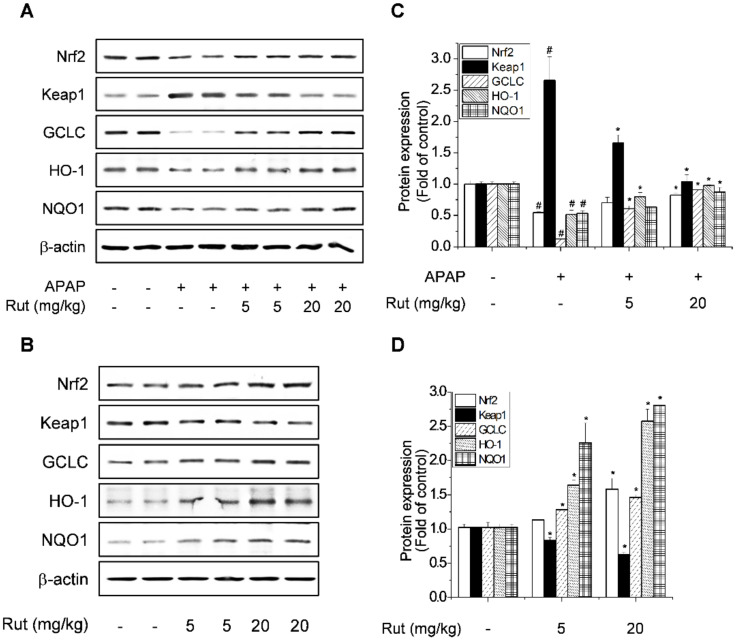
Protective effect of Rut as seen by the mitigation of the APAP-mediated reduction in antioxidant enzymes. Hepatic protein levels of Nrf2, Keap1, GCLC, HO-1, and NQO1 were determined using western blotting (**A**,**B**). Protein level was analyzed using ImageJ software. Relative expression of the target protein was compared using β-actin as a control (**C**,**D**). Results are indicated as means ± SD (*n* = 10). ^#^ Significantly different from the control (*p* < 0.05). * Significantly different from the APAP-treated group (*p* < 0.05).

GSH protects hepatocytes from the toxic effects of APAP, so we assayed the liver GSH level. Rut pretreatment prevented the APAP-mediated reduction in GSH levels (Figure 1D). Furthermore, Rut stimulated GSH biosynthesis, which is related to the expression of genes targeting Nrf2, so we explored the effect of Rut on the induction of Nrf2 target genes in APAP-treated mice. GCLC, HO-1, and NQO1 are important cellular antioxidant enzymes, the expression of which is regulated by Nrf2 [19], while Nrf2 is reportedly activated by Keap1. Nrf2 is normally sequestered in the cytoplasm by Keap1 but is degraded by oxidative stress. The effect of Rut on the APAP-mediated reduction in the expression of Nrf2-mediated antioxidant enzymes was determined using western blotting. The expression of Nrf2 and its target downstream genes, GCLC, HO-1, and NQO1, decreased as a result of the increased Keap1 degradation induced by APAP. These effects of APAP were reversed by Rut pretreatment (Figure 5A,C). The induction of antioxidant enzymes is a universal response to liver regeneration and exerts a hepatoprotective effect. Rut pretreatment increased the expression of Nrf2 target genes in a dose-dependent manner but significantly decreased that of Keap1 (Figure 5B,D). Therefore, Rut pretreatment protected against oxidative stress in APAP-treated mice, suggesting that its prevention of APAP-induced hepatic injury is mediated by the upregulation of Nrf2, which then activates the antioxidant enzyme pathway.

### 3.4. Rut Pretreatment Attenuated APAP-Induced Hepatotoxicity by Inhibiting JNK1/2 Signaling

**Figure 6:**
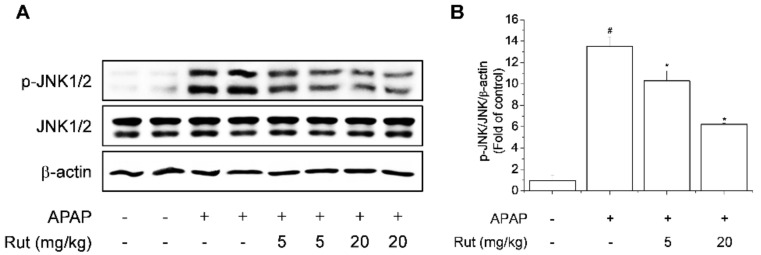
Protective effect of Rut on APAP-induced JNK1/2 activation in mice. Hepatic protein levels of phospho-JNK1/2 and JNK1/2 (**A**) were determined by western blotting. Protein level was analyzed using ImageJ software. Relative expression of the target protein was compared using β-actin as a control (**B**). Results are indicated as means ± SD (*n* = 10). ^#^ Significantly different from the control (*p* < 0.05). * Significantly different from the APAP-treated group (*p* < 0.05).

Because the JNK1/2 signaling pathway is associated with APAP-induced hepatotoxicity, we next evaluated the influence of the JNK1/2 pathway on the protective effect of Rut in APAP-induced liver injury [20]. APAP significantly induced the phosphorylation of JNK1/2 but Rut pretreatment significantly suppressed APAP-induced phosphorylation of JNK1/2 in a dose-dependent manner (Figure 6A,B).

## 4. Discussion

Hepatoxicity can be induced by viral infection, excessive alcohol consumption, drugs, environmental pollutants, and other factors. Drug-induced toxicity is the main cause of acute liver damage, and APAP is most frequently implicated in overdose cases. Hepatic toxicity is a common pathological feature of many liver diseases and can lead to hepatitis, hepatic fibrosis, cirrhosis, and hepatic cancer [21]. Therefore, preventive strategies against liver damage are important for preventing or ameliorating liver diseases.

APAP is sold worldwide to reduce pain and fever. APAP has few side effects when taken at therapeutic doses, but overdose can lead to inflammation, hepatocellular injury, and liver failure. Indeed, impaired hepatic function resulting from APAP overdose is the most common cause of drug-induced liver damage worldwide [22]. APAP induces acute hepatotoxicity and is used in animal models to evaluate the hepatoprotective effect of natural agents [23]. Liver injury caused by APAP is known to cause severe liver damage from 3 h after APAP administration, worsening after 6 h, and leading to extensive hepatocyte death after 24 h in a mouse model [20]. Many herbal extracts and compounds have been studied for their protective effects in the early stages of APAP-induced hepatotoxicity [16,19,24]. Therefore, we evaluated the protective effects and the related mechanisms of Rut on APAP-induced acute liver injury.

APAP overdose resulted in severe hepatic damage characterized by a high level of hepatotoxicity as indicated by the serum ALT/AST level and hepatic MDA content in treated mice [25,26]. APAP-induced hepatotoxicity is initiated by the formation of NAPQI by CYP2E1. NAPQI is removed upon reacting with liver GSH, but when GSH is depleted, the reactive metabolites produced accumulate and bind to macromolecules, causing liver toxicity effects [27]. This causes hepatic dysfunction, leading to hepatocyte injury and acute liver damage. In addition, APAP induces hepatic structural damage and necrosis. Rut pretreatment inhibited ALT/AST release, MDA formation, GSH depletion, and histopathological changes, indicating amelioration of hepatocyte damage [16,28,29]. The mechanism underlying the protective effect of Rut in APAP hepatotoxicity was the inhibition of CYP2E1.

Overproduction of proinflammatory cytokines is a sign of liver damage, and inhibiting their production can restore liver function. Inflammatory responses are associated with the pathogenesis of hepatotoxicity due to APAP [21,30]. Proinflammatory cytokines are activated by the APAP metabolite NAPQI, leading to an inflammatory response. In addition, cytokines related to APAP-mediated hepatotoxicity are induced by NF-κB. NF-κB controls the expression of target genes that regulate inflammatory mediators, including IL-1β, IL-6, TNF-α, COX-2, and iNOS, which are associated with hepatotoxicity [30]. Thus, the suppression of NF-κB reduces inflammatory mediator-dependent liver injury. Our results showed that Rut pretreatment downregulated NF-κB activation and proinflammatory cytokine expression, indicating that the suppression of the NF-κB pathway is associated with the protective effect of Rut in APAP-induced liver damage.

The oxidative stress caused by APAP induces sustained activation of JNK, leading to liver damage and hepatocyte death due to the increased production of ROS in mitochondria. In APAP-induced hepatic damage, apoptotic hepatocytes release endogenous damage-associated molecular patterns to induce an inflammatory response, and the phosphorylation of JNK1/2 and IκBα activates signaling proteins [16,20,31]. Our results showed that Rut pretreatment inhibited JNK1/2 activation as well as IκBα phosphorylation and degradation, suggesting that it ameliorates oxidative stress.

Nrf2, a modulator of multiple signaling pathways, protects against oxidative stress-induced apoptosis and mitochondrial dysfunction and inhibits a variety of diseases by regulating downstream antioxidant genes, such as GCLC, HO-1, and NQO1 [28]. The antioxidant-related protective mechanisms of Nrf2 in APAP-induced liver injury are established. In addition, some natural agents suppress APAP-induced hepatotoxicity by enhancing the expression of Nrf2 target genes such as NQO1, HO-1, and GCLC [32]. In this study, Rut pretreatment significantly restored the expression of the Nrf2 target genes NQO1, HO-1, and GCLC in mice with APAP-induced hepatotoxicity. Activated Nrf2 separates from Keap1, a redox sensor, translocates to the nucleus, and binds to the ARE in the promoter region of antioxidant genes [28,33]. Rut significantly suppressed the expression of Keap1 and increased that of Nrf2 target genes by promoting Nrf2 nuclear translocation and increasing ARE luciferase activity in a concentration-dependent manner in HepG2 cells [12]. Therefore, Rut pretreatment increases the Nrf2-mediated expression of antioxidant genes to attenuate APAP hepatotoxicity.

## 5. Conclusions

**Figure 7:**
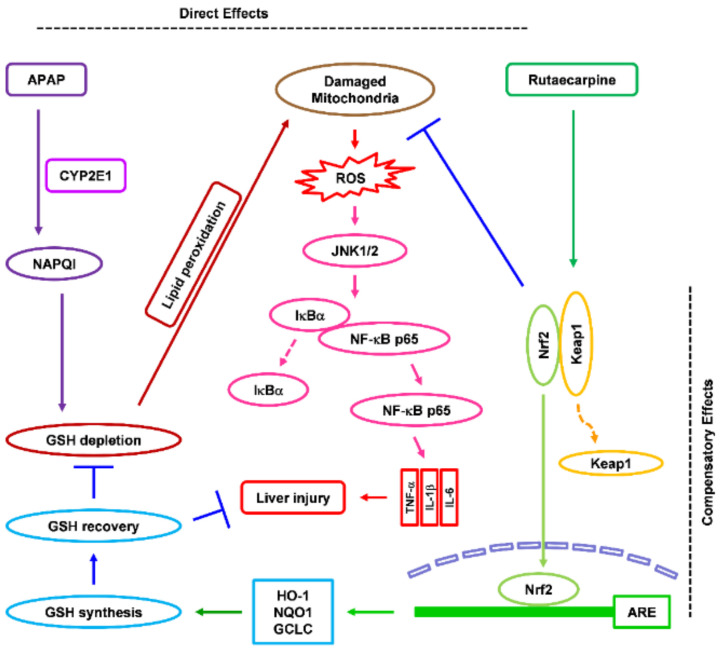
Protective effect of Rut in APAP-induced liver damage in mice. Direct effect. APAP is converted by CYP2E1 to NAPQI, a toxic metabolite. NAPQ1 depletes intracellular GSH and damages mitochondria by binding to mitochondrial proteins. Rut pretreatment inhibits the expression of CYP2E1, ameliorating intracellular GSH depletion, and inhibits lipid peroxidation, thereby preventing APAP-mediated mitochondrial damage. Compensatory effect. Rut pretreatment increases the nuclear translocation of Nrf2 by alleviating its inhibition by Keap1, causing sustained activation of Nrf2 in the mouse liver. Prolonged activation of Nrf2 increases the GSH content in the mouse liver, eliminating NAPQI and protecting the liver against APAP-induced oxidative stress.

In conclusion, Rut pretreatment ameliorates APAP-induced hepatic damage by inhibiting oxidative stress and liver inflammation by upregulating Nrf2-related antioxidant pathways (Figure 7). The results suggest that Rut has preventive potential against hepatotoxicant-induced liver damage.

## Figures and Tables

**Table 1:** Primer sequences for real-time PCR.

Gene		Sequences	NCBI Number
TNF-α	F	TTGTCTACTCCCAGGTTCTCTT	NM_001278601.1
	R	ACTTTCTCCTGGTATGAGATAGC	
IL-1β	F	GAAAGAATCTATACCTGTCCTGTGTAA	NM_008361.4
	R	CTTCTATCTTGTTGAAGACAAACCG	
IL-6	F	ATACAGAAACTCTAATTCATATCTTCAACC	NM_031168.2
	R	AGCTTATCTGTTAGGAGAGCAT	
β-actin	F	CCACCAGTTCGCCATGGAT	NM_007393.5
	R	CCACGATGGAGGGGAATACA	

## Data Availability

The data presented in this study are available on request from the corresponding author.
